# Frugivore Behavioural Details Matter for Seed Dispersal: A Multi-Species Model for Cantabrian Thrushes and Trees

**DOI:** 10.1371/journal.pone.0065216

**Published:** 2013-06-11

**Authors:** Juan Manuel Morales, Daniel García, Daniel Martínez, Javier Rodriguez-Pérez, José Manuel Herrera

**Affiliations:** 1 Laboratorio Ecotono, INIBIOMA-CONICET, Universidad Nacional del Comahue, Black River, Argentina; 2 Department Biología de Organismos y Sistemas, Universidad de Oviedo, and Unidad Mixta de Investigación en Biodiversidad (UMIB, CSIC-UO-PA), Oviedo, Spain; University of Zurich, Switzerland

## Abstract

Animal movement and behaviour is fundamental for ecosystem functioning. The process of seed dispersal by frugivorous animals is a showcase for this paradigm since their behaviour shapes the spatial patterns of the earliest stage of plant regeneration. However, we still lack a general understanding of how intrinsic (frugivore and plant species traits) and extrinsic (landscape features) factors interact to determine how seeds of a given species are more likely to be deposited in some places more than in others. We develop a multi-species mechanistic model of seed dispersal based on frugivore behavioural responses to landscape heterogeneity. The model was fitted to data from three-years of spatially-explicit field observations on the behaviour of six frugivorous thrushes and the fruiting patterns of three fleshy-fruited trees in a secondary forest of the Cantabrian range (N Spain). With such model we explore how seed rain patterns arise from the interaction between animal behaviour and landscape heterogeneity. We show that different species of thrushes respond differently to landscape heterogeneity even though they belong to the same genus, and that provide complementary seed dispersal functions. Simulated seed rain patterns are only realistic when at least some landscape heterogeneity (forest cover and fruit abundance) is taken into account. The common and simple approach of re-sampling movement data to quantify seed dispersal produces biases in both the distance and the habitat at which seeds arrive. Movement behaviour not only affects dispersal distance and seed rain patterns but also can affect frugivore diet composition even if there is no built-in preference for fruiting species. In summary, the fate of seeds produced by a given plant species is strongly affected by both the composition of the frugivore assemblage and the landscape-scale context of the plant location, including the presence of fruits from other plants (from the same or different species).

## Introduction

The role of animals as vectors linking ecological processes between habitat patches across space and time [1] derives from the idea that their movement and behaviour cascade into key ecosystem functions [2], [3]. Among these processes, seed dispersal by frugivorous animals is is perhaps the best studied and most emblematic. Decisions of frugivores on what to eat and where to move determine how seeds are deposited in space [4] and, hence, may ultimately drive the dynamics of plant populations and communities [5], [6]. In principle, it is possible to mechanistically predict seed dispersal based on frugivore behaviour and physiology (reviewed in [13]). However, we still lack a general understanding of how intrinsic (fruit and frugivore species traits) and extrinsic (landscape features) factors interact to determine whether seeds of a given species are more likely to be deposited in some places rather than in others.

The standard approach is to combine observed animal movement distance distributions with gut passage time of seeds in order to estimate dispersal kernels describing how the probability of seed arrival changes with distance to the plant of origin. However, frugivore movement is highly contingent on the abundance and distribution of fruit resources [7], [8], [9] and other landscape features such as the availability and arrangement of different habitats [10], [11], [12]. Ignoring these effects of landscape heterogeneity in animal movements could lead to oversimplified views of seed dispersal, implying very different demographic projections for plant populations [9], [13], [14] than those empirically experienced in real systems.

Most studies so far deal with single frugivore-plant pairs, ignoring the actual diversity of frugivore-fruit assemblages [15], [16]; but see [17], [18]. However, the fact that different plant species within a community usually share a common assemblage of frugivores introduces two additional sources of complexity into seed dispersal processes. First, different frugivores usually show strong differences in diet (e.g. [19], [20]), gut passage time (e.g. [17], [18]), and post-feeding movement behaviour (e.g. [21], [22]), resulting in high functional variability within frugivore guilds. Second, plant species may affect each other by contributing, depending on the spatial distribution of their fruiting adults, to generate community-wide fruiting landscapes to which frugivores are sensitive (e.g. [23], [24]). Disentangling the actual role of these two sources of complexity is challenging and requires the development of mechanistic models anchored to field data of both fruit distribution and frugivore behaviour at the landscape scale.

Here we develop a multi-species mechanistic model to explore how seed dispersal kernels and seed rain patterns arise from the interaction between animal behaviour and landscape heterogeneity. The model was fitted to data from three-years of spatially-explicit field observations on the behaviour of six frugivorous bird species and the fruiting patterns of three fleshy-fruited trees in temperate secondary forests of the Cantabrian Range [23]. In particular, we show that:

1. Bird species differ in their responses to landscape heterogeneity (large scale variability in forest cover and fruit availability).
2. Simulated (predicted) seed rain patterns are only realistic when at least some landscape heterogeneity is taken into account. The common and simple approach of resampling movement data to quantify seed dispersal produces unrealistic patterns both with regard to distance properties and the habitat at which seeds arrive.
3. Movement behaviour not only affects dispersal distance and seed rain patterns but can also affect bird dietary composition even if there is no built-in preference for different fruiting species.

From these facts it can be concluded that the fate of a seed is strongly affected by the context of the mother plant location, including the presence of fruits from other plants (from the same or different species). It is clear that realistic representations of frugivore-meditated dispersal should consider both movement biases and the composition of the frugivore assemblage.

## Methods

### Study System

We focused on the frugivore-plant system composed of thrushes (*Turdus* spp.) and fleshy-fruited trees in the temperate secondary-growth forest of the Cantabrian range, N Spain (see Text S1 for a detailed description). Frugivory and seed dispersal by thrushes has been shown to affect tree regeneration by triggering the processes of re-colonization of deforested areas [23], and by driving the patterns of long-term recruitment at different spatial scales [25], [26]. Other small birds (e.g. Blackcap *Sylvia atricapilla*) and carnivorous mammals (e.g. Red Fox *Vulpes vulpes*) are able to disperse the seeds of fleshy-fruited plants in the Cantabrian range, but, they only feed occasionally on fruits of secondary forest trees [27].

The studied thrushes, ordered by increasing size (see Table S2.1 in Text S2), were Redwing *T.iliacus* L., Song Thrush *T. philomelos* Brehm, Blackbird *Turdus merula* L., Fieldfare *T. pilaris* L., Ring-Ouzel *T. torquatus* L. and Mistle Thrush *T. viscivorus* L. Among them, *T. iliacus, T. pilaris* and *T. torquatus* are over-wintering species in northern Spain, whereas *T. philomelos*, *T. merula*, and *T. viscivorus* are resident species that receive overwintering migrants [28]. All thrushes are “insectivorous” birds whose diet turns to almost exclusively frugivore during autumn and winter. They swallow the entire fruits, defecating (and occasionally regurgitating) the intact seeds in their faeces and, thus, acting as legitimate seed dispersers [29].

The main fleshy-fruited tree species dispersed by thrushes are Holly (*Ilex aquifolium* L.), Hawthorn (*Crataegus monogyna* Jacq.), Yew (*Taxus baccata* L.), which account for a large proportion of tree cover in the Cantabrian secondary forests under study (ca. 70%, e.g. [25]. Fruits of these species are sugar-rich red berry-like fruits (arillated seed in yew), 10–14 mm in diameter, and contain 1–4 seeds (5–9 mm). All species ripen in autumn. In sum, by focusing here on the interactions between dominant fruiting tree species (*I. aquifolium, C. monogyna and T. baccata*) and thrushes, we are considering an important fraction of the whole plant-frugivore assemblage accounting for a large proportion of tree-community seed dispersal service.

### Field Study

The field study area was located in the Sierra de Peña Mayor (43° 17′N, 5° 30′W, 900 m a.s.l., Asturias Province, Spain; see Text S1 for a detailed description of study site and field methodologies). Field work was done under permission of the Consejería de Medio Ambiente (Regional Government of Asturias).We set up a 400×440 m rectangular plot in which the amount of forest cover varied from densely covered sectors to areas of scant cover and isolated remnant trees (see Fig. S1.1 in Text S1). For sampling purposes, the plot was subdivided into 440, 20×20 m cells where we estimated the amount of forest cover (in m^2^, and irrespective of tree species identity) in each cell. The size and resolution of the plot matches the scales of rutinary activities of the thrushes [30]. Field sampling was carried out from September to February along three consecutive sampling periods: 2007–2008, 2008–2009 and 2009–2010 (hereafter 2007, 2008 and 2009).

In October of each sampling year, we mapped all individual trees (>1.5 m tall or 4 cm trunk basal diameter) within the plot, assigning visually a standing crop to each individual of any fleshy-fruited species by means of a Fruiting Abundance Index (considering six intervals: 0 = without fruits; 1 = 1–10 fruits; 2 = 11–100; 3 = 101–1,000; 4 = 1,001–10,000; 5≥10,001). We calculated the total abundance of fruits per landscape cell, and the abundance of fruits of different tree species per year as the sum of the crops of all fruiting trees present each year.

Foraging patterns of thrushes were sampled over observation sequences made from five different vantage positions in elevated outcrops (hill tops), located along the central axis of the plot (Fig. S1.1 in Text S1). Sampling season extended from October to February. Observation time was 78, 90 and 79 hours for 2007, 2008 and 2009 respectively. In each sequence, a movement bout of a given individual bird was tracked with the help of 8×30 binoculars, a chronometer, and printed maps of plot cells. Once a given focal bird was located, it was followed until lost, either because it left the plot or disappeared into the canopy. For all sequential steps in the movement bout (i.e. consecutive rests separated by intervening flights), we recorded the duration and location of the resting site (i.e. the cell within the plot), and the species and number of fleshy fruits eaten while perching in a fruiting tree. Flight distance was calculated for each flight between rests located in different cells as the Euclidean distance between the centroids of the starting point and endpoint cells. All observers were well trained on bird identification and observation, and their observation time was allocated across years and watching positions in order to avoid potential biases due to disproportionate observation of a given observer from a given position and for a given year.

We assessed the occurrence of seeds dispersed by thrushes in sampling stations across the whole plot in 2009. Ten sampling stations, separated from each other by 2 m, were placed along the central longitudinal axis of 220 sampling cells following a checkered pattern (see Fig. S1.1 in Text S1). In each station, we set up a permanently labelled, open-ground 50×50 cm quadrat where all tree seeds dispersed by birds were collected and counted. We estimated the number of dispersed seeds per tree species and sampling station as the sum of seeds found in two consecutive surveys (late November 2009 and early January 2010).

### Model Development

We adapted Morales and Carlo simulation model [7] in order to be able to parameterize the movement rules of dispersal agents based on the available data. The model is a spatially explicit, event driven, stochastic simulation of bird foraging and seed dispersal which has been used so far as a tool for theoretical studies of the interplay between animal behaviour and plant spatial structure [9], [13]. An important difference between the Morales and Carlo model and the one we use here is that, given the nature of our data, bird movement is simulated as going from landscape cell to landscape cell rather than from plant to plant. Below we describe the general simulation structure and how we parameterize each component.

### Gut Passage Time

Every frugivory event was tied to a gut passage time (GPT) drawn from a Gamma distribution with a common shape parameter but with a bird species-specific scale parameter. As no empirical data on GPT was available from any of the studied bird-fruit pairs, we fitted a Gamma distribution to data from *Turdus merula* eating fruits of *Myrtus communis* (Mar Sobral, Asier R. Larrinaga & Luis Santamaría, unpublished data). GPT is known to depend on bird size [31] and hence for the purpose of our simulations we calculated expected values for GPT for the other five *Turdus* species based on a linear model fitted to published data on mean GPT from 8 species from Turdidae and Sylviidae [31]. Details can be found in Text S2.

### Residence Time and Fruit Consumption

Simulated birds spent a variable amount of time grounded or perching at a particular location. Every time a simulated bird arrived at a landscape cell, the time spent before making a new movement decision was a random value drawn from a Gamma distribution fitted to the observed perching time for each species (Text S3). Time perching was independent from fruit consumption as in the original model formulation from Morales and Carlo [7]. In a total of 705 opportunities, observers in the field were able to clearly see whether a focal bird consumed fruits or not. There was no correlation between time spent at a plant and the number of fruits consumed (Pearson’s *r* = 0.04, *t* = 1.07, *df* = 703, *P* = 0.28). Separate analyses for each species were also not significant (Table S3.2 in Text S3).

To model fruit consumption we considered both observed consumption rates and fruit availability at the landscape cell. Potential fruit consumption was drawn from a Zero-inflated Poisson distribution fitted to the observed number of fruits consumed by each *Turdus* species irrespective of plant species or other factors (Table S3.3 in Text S3). The model then assigned a plant species identity to fruits consumed during a particular feeding bout by drawing a species name with probabilities given by the species abundance at the landscape cell (i.e. there is no built-in preference for fruit species in the model). The simulated fruit consumption was the minimum between this number and the number of fruits available in the landscape cell. After a frugivory event, the number of fruits in the landscape cell was immediately updated.

### Bird Movement

Movement decisions by the simulated birds were made once perching time expired. An individual could choose to stay in the same landscape cell, move to another cell, or leave the study plot. The program computed first the probability (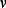) of leaving the study plot as a function of distance to the nearest edge (*B*):
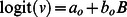
(1)


Where *a_o_* and *b_o_* are parameters fitted to bird movement data. When moving within the study plot, we assumed that three main factors affected where a bird decided to go: (1) distance to current location, (2) forest cover and (3) the abundance of fruits at the potential destination. With these factors, we built a discrete probability distribution as follows:
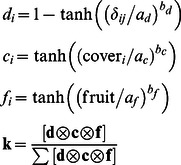
(2)


Here **d**, **c** and **f** are vectors of length 440 (one value for every landscape cell in the study plot) that hold the relative probability of choosing the *i*-th landscape cell given distance to current location, forest cover and fruit abundance respectively. The hyperbolic tangent, tanh(*x*) is a sigmoid function that takes values between -1 and 1 with inflection at *x = *0. The scale parameters 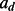, 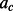 and 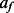 control how quickly the relative probabilities increase or decrease with changes in distance, cover and fruit availability, while the shape parameters 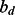, 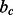 and 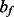 determine the form of such changes. The elements of vectors **d**, **c** and **f** are multiplied (Kronecker product 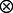
in eq. 2) and the resultant vector standardized into a discrete probability distribution **k**.

The calculations described in eq. 1 and 2 above were used first to compute likelihood functions to estimate parameters from observed trajectories and later-on to build probability distributions that were sampled in order to simulate bird movement decisions with the fitted parameter values. Using the available data for every species and the fruit abundance data from every year, we tried different combinations of factors (distance only, distance plus fruit, distance plus cover and distance plus cover and fruit) and selected the most relevant combination based on AICc values. Details on parameter values and standard errors can be found in Table S4.1 in Text S4.

Once a simulated bird decided where to go, it flew at a speed of 6 meters per second [32] following a straight line between current location and destination. The simulation program kept track of the time at which each consumed seed should stop travelling with the animal. The location of the bird at each defecation event was found and the corresponding landscape cell was recorded as the destination for those dispersed seeds. The identity of the landscape cell where the mother plant was located was also recorded.

### Simulations

In order to quantify the importance of movement decisions on seed rain, we run a “full model” were every bird species moved according to parameters fitted to observed trajectories, and a “distance only” version where bird movement was sampled with replacement from the observed data. The simulations were run over the landscape cover map and using fruit abundance data from 2009.

For each model, we simulated 5 thousand bird movement sequences for 30 replicates. Simulated birds were introduced in the landscape sequentially, with their species identity sampled from the relative abundance observed in 2009 (see [33] for a complete description of the method used to estimate bird abundances). The initial location was chosen at random and the simulated bird moved, perched, foraged and dispersed seeds following the rules described above until it left the study plot. Data from the first five moves were discarded to minimize the effect of initial locations (discarding more moves did not change the results).

### Spatial Analysis

Once the predicted number of dispersed seeds from the different tree species by each of the species of thrushes was obtained through simulations, we were interested in evaluating how the seed rain generated by each bird was shaped by landscape features, i.e. forest cover and fruit availability. We thus built Spatial Simultaneous Autoregressive Models (SAR; [34] considering as a response variable the proportion of seeds accounted by each cell (from the total number of seed dispersed, arcsin sqrt) and, as predictor variables, forest cover (arcsin sqrt proportion) and fruit abundance (log number of fruits per cell). We run similar models for both the proportion of seeds dispersed predicted by the full model and that predicted by the distance-only model.

We also sought to assess the ability of the simulation model to predict the large-scale spatial structure of seed dispersal observed in the field. For this, we first estimated the degree of fit between predicted and observed seed dispersal patterns. We used the Spatial Analysis by Distance Indices (SADIE; [35]), a method that describes the spatial structure of ecological data sampled in the form of spatially geo-referenced counts (i.e. number of dispersed seeds), identifying and locating the areas where patches of high or low density occur (see a complete description in Text S5). We characterized the plot-scale spatial structure of predicted and observed seed rain of the different tree species by quantifying, by means of an aggregation index (*Ia*), the degree of spatial aggregation (patchiness) in the abundance of dispersed seeds. Then, we estimated, by means of an association index (*Xp*), the strength of the spatial match between the distributions of observed and predicted seed rains.

Secondly, we compared observed and predicted seed rains in terms of response to landscape features (i.e. forest cover and fruit availability). We built up SAR models checking the relative effect of forest cover (arcsin sqrt proportion per cell) and fruit abundance (log number fruits per m^2^ per cell) in the abundance of dispersed seeds of each studied tree species, for both observed seed rain (log seeds per m^2^ per cell) and predicted seed rain (log seeds per cell; *N* = 220 cells).

## Results

### Bird Observations and Model Parameterization

**Figure 1:**
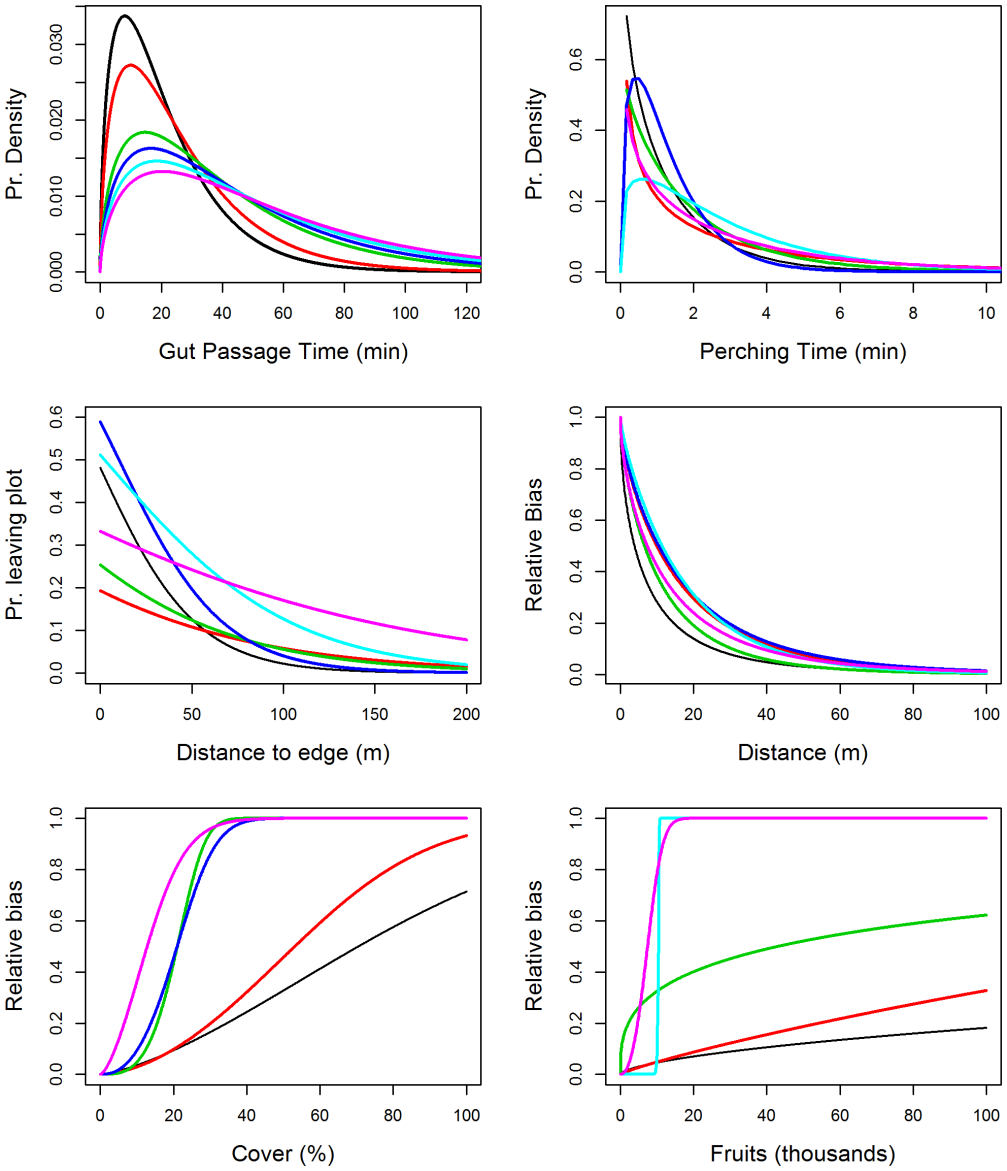
Model functions fitted to different ***Turdus*** **species.** Gut passage time (A) is Gamma distributed with scale parameter related to bird size. Perching time (B) is Gamma distributed and fitted to data from direct observations. The probability of leaving the study plot (C) decreased with distance to the edge. Movement to a landscape cell decreased with distance (D). Movement bias increased with forest cover and with fruit abundance (E and F). The *Turdus* species are: *T. iliacus* (black), *T. philomelos* (red), *T. merula* (green), *T. pilaris* (blue), *T. torquatus* (cyan) and *T. viscivorus* (magenta). No effect of fruit abundance was found for *T. pilaris* and no effect of cover for *T. torquatus*.

We recorded a total of 656 tracks belonging to six species of thrushes. On average, tracks lasted 2.87 moves (range 1 to 13). Perching time distributions were characterized by frequent brief periods with occasional extended periods (Fig. S3.1 and Table S3.1 in Text S3). Median perching time tended to increase with bird body size (Fig. 1B, Pearson’s product moment correlation *r* = 0.91, *t* = 4.29, *df* = 4, *P* = 0.013). Birds consumed fruits during roughly half of the instances in which they were perching at fruiting trees and the number of fruits ingested per feeding bout was usually between four and eight with no apparent relation to body size (Table S3.3 in Text S3).

Different bird species reacted differently to distance, forest cover and fruit abundance (Fig. 1D toF). For the less abundant *T. pilaris* and *T. torquatus* model selection favoured the exclusion of either fruit abundance or cover as factors affecting movements (Table S4.2 in Text S4). We also compared the distributions of observed bird displacements to simulated ones. Overall, observed and simulated movement distance distributions were similar although simulations tend to have a higher frequency of long moves (Fig. S4.1 in Text S4).

In general, birds tended to make short moves with occasional long ones. They biased their movements towards areas with more forest cover and fruit abundance. The larger species (notably *T. viscivorus*) were more likely to make longer moves and to leave the observation area even if they were located well inside the study plot (Fig. 1Cand D). Both *T. iliacus* and *T. philomelos* increased their movement bias gradually as forest cover increased while *T. merula*, *T. pilaris* and *T. viscivorus* responded strongly at low values of forest cover, saturating their bias roughly around 40% cover (Fig. 1E).

Regarding fruit abundance, *T. iliacus* and *T. philomelos* increased their movement bias gradually (almost linearly) with increasing fruit abundance with *T. philomelos* having a larger slope (Fig. 1F, black and red lines respectively). *T. merula* showed a sharp increase in movement bias when fruit abundance increased from none to a few thousand and then continued to increase gradually (Fig. 1F, green line). In contrast, both *T. torquatus* and *T. viscivorus* seem to have something close to an “all-or-nothing” response to fruit abundance, sharply increasing and saturating their movement bias with once fruit is present in a landscape cell (Fig. 1D, thick cyan and magenta lines respectively).

### Simulation Results

**Figure 2:**
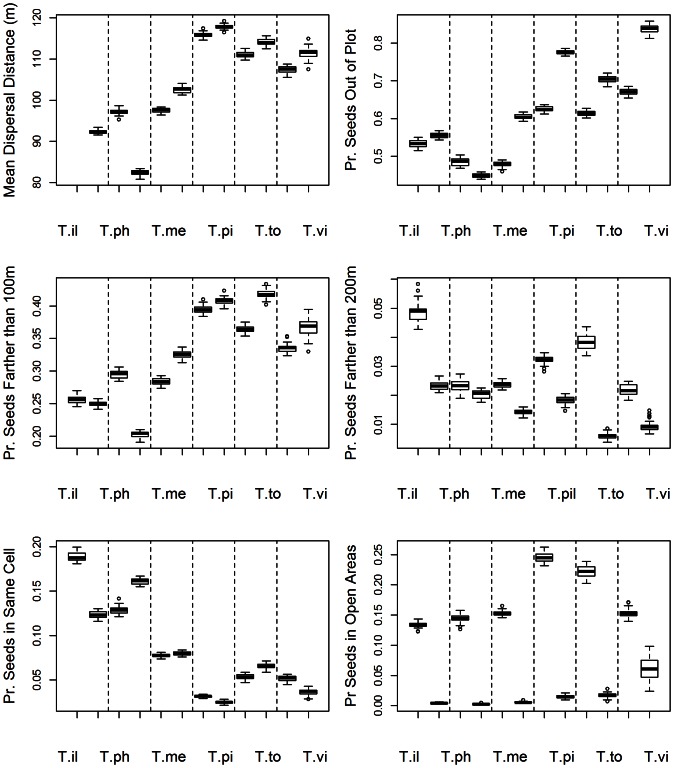
Distance properties of seed shadows by different ***Turdus*** **species.** Every pair of boxplots within dashed lines corresponds to the results from 30 replicate model runs for a particular *Turdus* species. The first box is for a “distance-only” model and the second one for the “full” model. When seeds were dispersed out of the study plot, we used as dispersal distance the value just outside the nearest edge. Bird species are *T. iliacus* (T. il), *T. philomelos* (T. ph), *T. merula* (T. me), *T. pilaris* (T.pi), *T. torquatus* (T. to) and *T. viscivorus* (T. vi).

**Figure 3:**
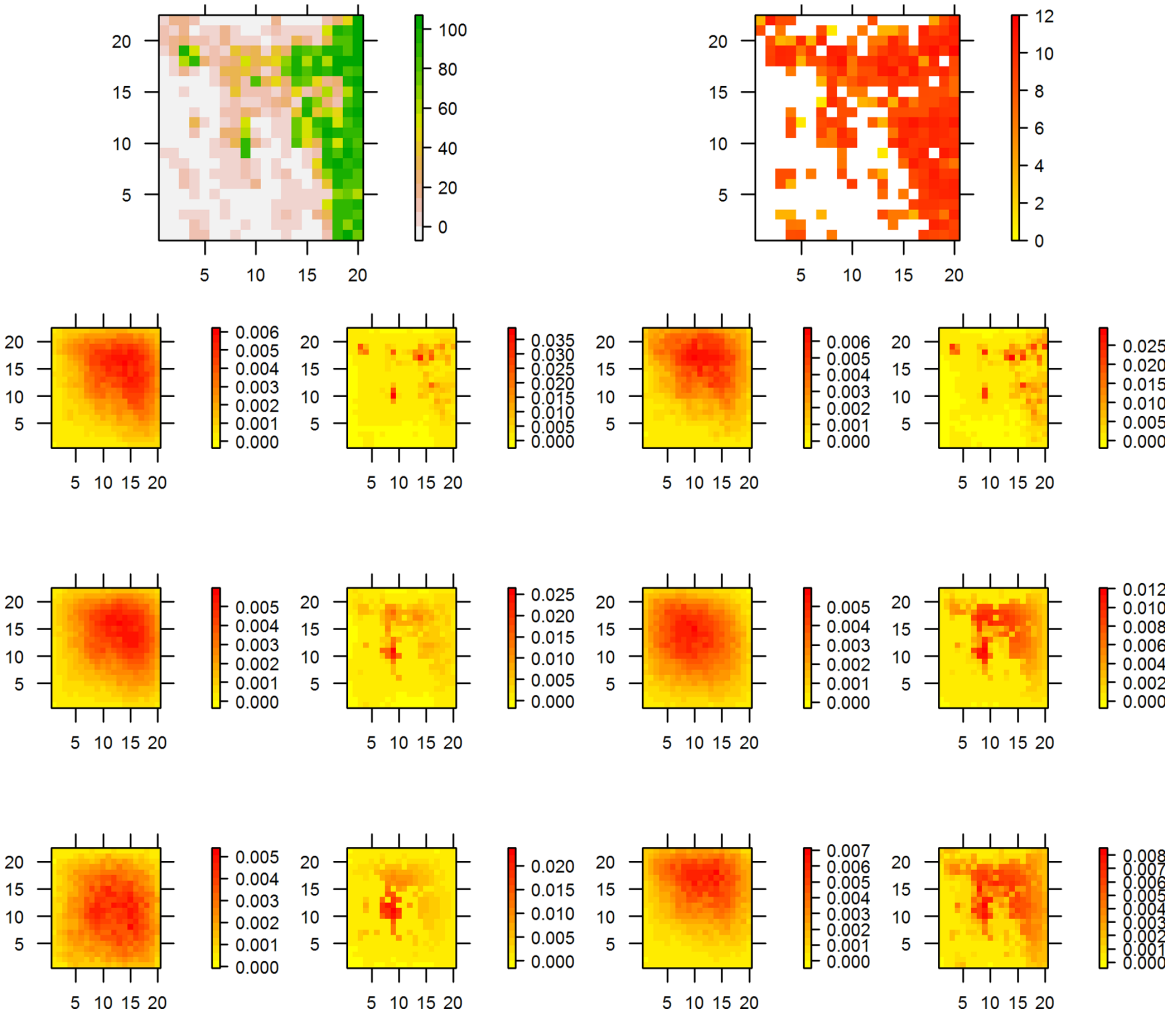
Cover, fruit abundance and maps of simulated seed rain by each bird species. Seed rain maps hold the proportion of seeds dispersed to each landscape cell by each bird species. The left column under each species is for a “distance-only” model and the right one for the “full” model including biases due to forest cover and fruit abundance.

The species-specific differences in movement and foraging patterns translated into different dispersal distances and spatial patterns of seed deposition (Figs. 2 and 3). The fact that birds biased their movement according to forest cover and fruit abundance had a strong effect on both seed dispersal distances and seed rain patterns. The naïve approach of re-sampling observed movement (or running simulations with distance as the only factor) resulted in very different predictions compared to the full model. Including movement bias due to fruit abundance and forest cover generally predicted shorter mean dispersal distances (Fig. 2A) but more seeds dispersed out of the study plot (Fig. 2B). Dispersal at relatively large distances (100 and 200 m) did not show consistent differences between the distance-only and the full model but there was a tendency for the full model to have smaller probabilities of long distance dispersal (Figss 2C and D). The full model also resulted in more seeds dropped in the landscape cell of origin (Fig. 2E) and in an important reduction in the proportion of seeds dispersed into landscape cells with no forest cover (Fig. 2F).

Predicted seed rain patterns were much more homogenous under the distance-only model compared to the full model where the effects of forest cover and fruit abundance were clearly visible (Fig. 3). Each *Turdus* species produced different seed rain patterns, resulting from different responses to forest cover and fruit availability (Table 1; Fig. 3). For example, *T. iliacus* and *T. philomelos* generated seed rains that responded strongly to forest cover distribution across the plot, whereas *T. viscivorus* generated a more widespread seed rain across the whole plot.

**Table 1:** Spatial Simultaneous Autoregressive Models (SAR) evaluating the relative effect of forest cover (arcsin sqrt proportion per cell) and fruit abundance (log number fruits per cell) in the proportion of seed dispersed per cell (from the total number of seed dispersed, arcsin sqrt) for the different species of frugivorous thrushes.

		Full Model Seed Rain				Distance-only Model Seed Rain			
Species	Factor	*R^2^*	Std. SAR Coefficient	*t*	*P*	*R^2^*	Std. SAR Coefficient	*t*	*P*
*Turdus iliacus*	Forest cover	0.687	0.778	16.79	<0.001	0.286	0.231	6.99	<0.001
	Fruit abundance		0.147	3.46	<0.001		0.210	6.93	<0.001
*Turdus philomelos*	Forest cover	0.777	0.787	19.46	<0.001	0.166	0.052	1.13	0.258
	Fruit abundance		0.162	4.32	<0.001		0.259	6.15	<0.001
*Turdus merula*	Forest cover	0.571	0.403	8.01	<0.001	0.189	0.204	5.61	<0.001
	Fruit abundance		0.474	10.29	<0.001		0.157	4.71	<0.001
*Turdus pilaris*	Forest cover	0.451	0.385	6.73	<0.001	0.008	−0.035	−0.54	0.588
	Fruit abundance		0.343	6.54	<0.001		0.061	1.03	0.305
*Turdus torquatus*	Forest cover	0.331	−0.076	−1.14	0.256	0.016	0.018	0.24	0.812
	Fruit abundance		0.672	10.95	<0.001		0.092	1.31	0.192
*Turdus viscivorus*	Forest cover	0.492	0.247	4.74	<0.001	0.127	0.062	1.32	0.186
	Fruit abundance		0.465	9.73	<0.001		0.207	4.82	<0.001

For each species, SAR are applied to the proportion of dispersed seeds estimated from the seed number predicted by both a full model and a distance-only model (*N* = 440 cells; R^2^ indicates the proportion of variance of the response variable explained by predictors without space effect).

**Figure 4:**
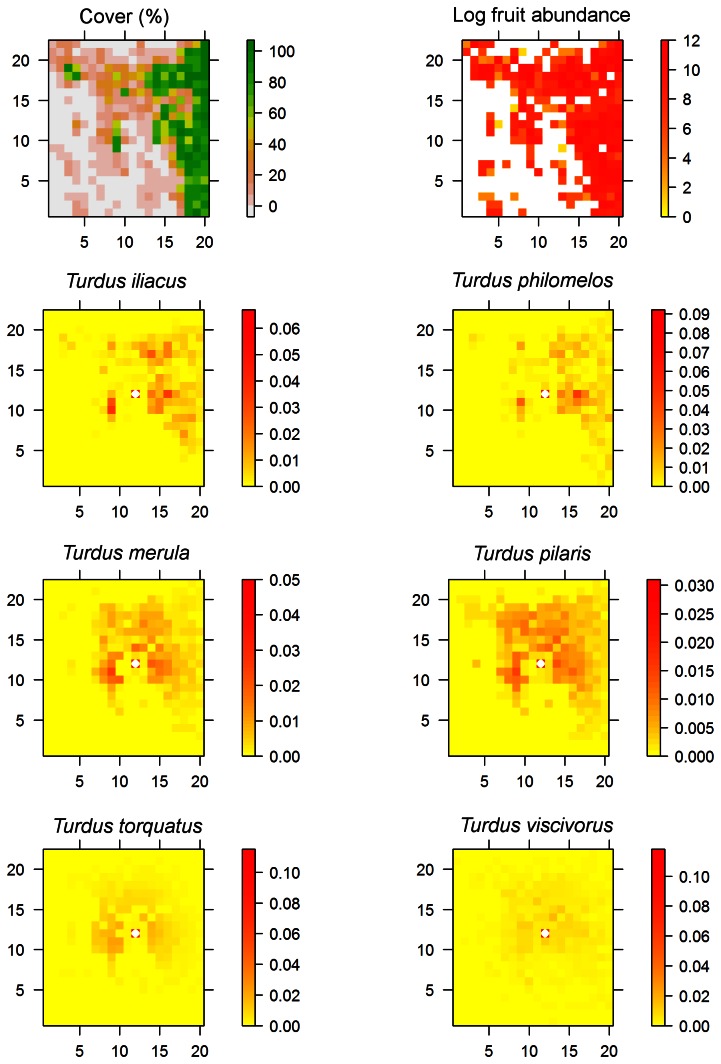
Cover, fruit abundance and maps of simulated seed rain from a central landscape cell (marked with a white dot) by each bird species. Seed rain is mapped as proportion of the total number of seeds dispersed.

Between species differences and the importance of cover and fruit abundance were also apparent when looking at the seed shadows of focal landscape cells (Fig. 4). Seed shadows were clearly biased due to spatial heterogeneity in forest cover and fruit abundance. The smaller species *T. iliacus* and *T. philomelos* were sensitive to both landscape features while *T. merula* was more sensitive to fruit abundance than to forest cover (Fig. 1 E and F, Fig. 4). There was a marked difference in the seed rain generated by *T. pilaris* who responded to forest cover but ignored fruit abundance and *T. torquatus* who did the opposite. Finally, *T. viscivorus* was not very sensitive to differences in forest cover and fruit abundance beyond some moderate values (Fig. 1 E and F) and this resulted in a more homogeneous seed rain compared to the other species (Fig. 4).

The interactions between bird behaviour and landscape properties are also apparent when looking at dispersal distances for each pair of plant and bird species. As plant species are not distributed homogeneously across the study site and birds react to the distributions of both forest cover and fruit availability, our model predicts that, in general, a particular bird species will generate different dispersal distances for each of the three plant species (Table 2).

**Table 2:** Mean dispersal distances produced by each *Turdus* species varied among fleshy-fruited plant species reflecting landscape effects on bird behaviour.

	Crataegus monogyna		Ilex aquifolium		Taxus baccata	
*T. iliacus*	89.47	(87.80–90.72)	80.65	(80.26–81.38)	68.41	(65.56–69.99)
*T. philomelos*	81.57	(79.08–83.12)	72.44	(71.66–72.89)	61.05	(59.82–62.91)
*T. merula*	87.17	(85.79–88.02)	82.05	(81.49–82.68)	86.33	(82.49–89.06)
*T. pilaris*	113.54	(112.46–115.16)	109.11	(107.96–109.91)	117.07	(108.86–122.07)
*T. torquatus*	79.19	(77.69–81.22)	77.63	(76.58–78.49)	84.14	(77.58–89.14)
*T. viscivorus*	94.06	(90.33–98.24)	89.45	(87.57–91.72)	85.67	(72.68–96.58)

When seeds were dispersed out of the study plot, we used as dispersal distance the value just outside the nearest edge. Values within brackets correspond to 95% intervals for the distribution of dispersal distances.

**Figure 5:**
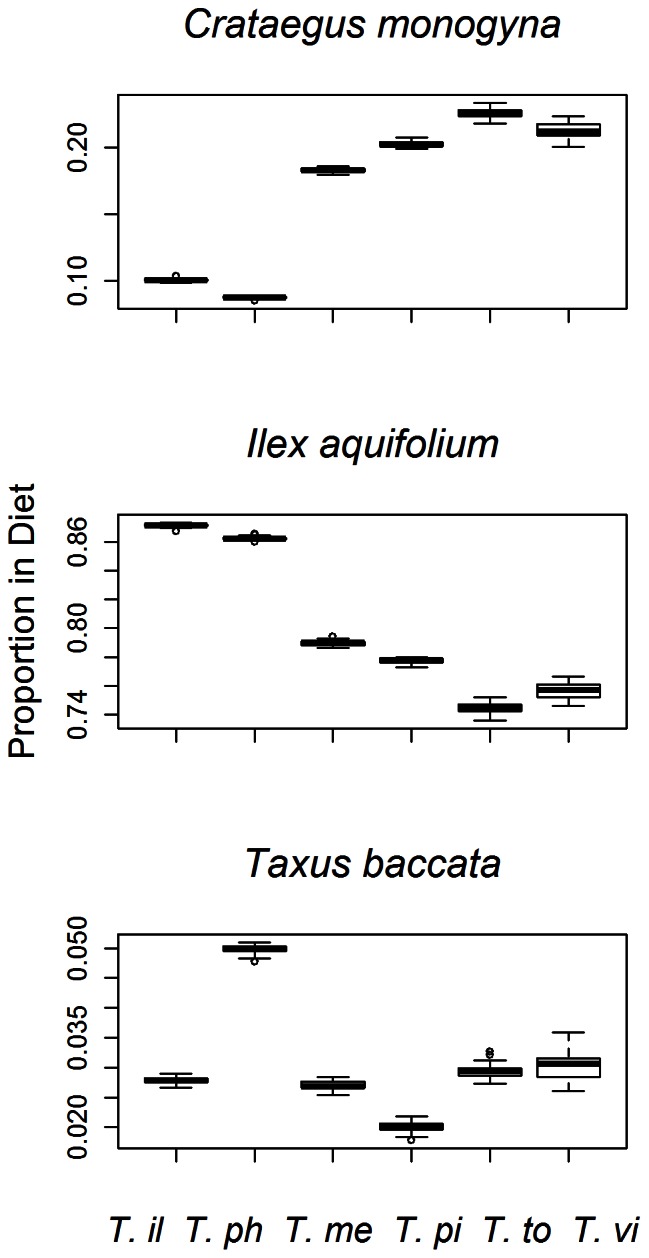
Different ***Turdus*** **species had different diets despite the fact that no preference was built-in in the model.** Bird species are *T. iliacus* (T. il), *T. philomelos* (T. ph), *T. merula* (T. me), *T. pilaris* (T.pi), *T. torquatus* (T. to) and *T. viscivorus* (T. vi).

Bird movement rules not only affected dispersal distances and seed rain patterns but also their diet. Interestingly, we found that different bird species encountered species of fruit at different rates because they moved at different spatial scales and reacted differently to forest cover and fruit abundance. Despite the fact that our simulated birds had no built-in preferences for fruit species, there were detectable differences in fruit consumption (Fig. 5).

### Comparison with Seed Rain Data

The SADIE-derived aggregation indexes of both observed and predicted abundances of dispersed seeds indicated strong and significant patchiness (Text S5). Aggregation was stronger on *I. aquifolium* seed rain than on those of *C. monogyna* and *T. baccata*, in both the observed and predicted estimates. The seed rain predicted by the simulation model reproduced quite well the landscape-scale spatial structure of observed seed rain. For all studied tree species, there was a positive and significant spatial match between the landscape-scale patchiness of observed seed rain and that of seed rain predicted by the model, increasing from *C. monogyna* to *I. aquifolium* and *T. baccata* (as judged by the SADIE-derived association indexes, Table S5.2 in Text S5). Both the observed and predicted seed rain of all tree species responded in similar ways to landscape features measured in the field in 2009 (Table 3). SAR models indicated that *I. aquifolium* and *C. monogyna* seed deposition were significantly and positively affected by forest cover and fruit abundance, whereas *T. baccata* seed rain was exclusively affected by forest cover.

**Table 3:** Spatial Simultaneous Autoregressive Models (SAR) evaluating the relative effect of forest cover (arcsin sqrt proportion per cell) and fruit abundance (log number fruits per m^2^ per cell, from all fruiting species) on seed rain for both observed (log seeds per m^2^ per cell) and predicted seed rain (log seeds per cell; *N* = 220 cells; *R^2^* indicates the proportion of variance of the response variable explained by predictors without space effect).

		Observed Seed Rain				Predicted Seed Rain			
Species	Factor	*R^2^*	Std. SAR Coeff.	*t*	*P*	*R^2^*	Std. SAR Coeff.	*t*	*P*
*Crataegus monogyna*	Forest cover	0.283	0.380	4.66	<0.001	0.382	0.286	3.82	<0.001
	Fruit abundance		0.189	2.35	0.019		0.352	4.82	<0.001
*Ilex aquifolium*	Forest cover	0.594	0.451	6.43	<0.001	0.577	0.555	8.17	<0.001
	Fruit abundance		0.338	5.01	<0.001		0.171	2.63	0.009
*Taxus baccata*	Forest cover	0.303	0.604	6.69	<0.001	0.583	0.597	8.62	<0.001
	Fruit abundance		0.084	0.96	0.34		0.120	1.82	0.07

## Discussion

Seed dispersal by animals is a complex process in which several consecutive stages and their features (gut passage time, perching time, travelling times) interact with spatial processes (e.g., biases due to landscape features, the distribution of resources) to produce a wide range of seed rain patterns [9], [10], [11], [12]. Here, we have combined detailed landscape maps and direct observations of bird foraging behaviour and movement biases from six species of thrushes into a mechanistic model of seed dispersal. In this way we were able to model species-specific reactions to landscape heterogeneity and to predict seed dispersal patterns for three fleshy-fruited plant species sharing a frugivore assemblage. Thus, we show that the fate of seeds produced by an individual plant was strongly affected by both (i) the intrinsic traits of the frugivore- plant assemblage (i.e. frugivore guild composition), and (ii) the extrinsic features of the landscape-scale context, such as forest cover or the presence of fruits from other plants.

### Frugivore-Plant Assemblage and Seed Dispersal Patterns

Our simulation results evidence large differences between frugivore species, even though they all belong to the same *Turdus* genus. Previous observational studies suggest that behavioural particularities among thrushes (e.g., fruit consumption, microhabitat selection for perching, flight distances, large-scale spatial distribution; [23], [27]) may contribute to the generation of community-level seed dispersal patterns. Our work goes beyond these studies as it enables us to explain community-wide seed dispersal patterns as a sum of the specific contributions by the different frugivores, resulting from their responses to habitat cover and fruit availability (Fig. 1). In differing in the distance, the type of location (habitat) and the spatial extent over which they deposit seeds (Figs. 2 and 3), different thrushes complement each other in their seed dispersal functions (see also [16]).

The present study also highlights the influence of habitat availability and the community-wide fruiting environment on seed rain. Previous observational work has found strong effects of forest cover and fruit abundance on the patterns of seed arrival of some fleshy-fruited species [23], [36]. Here, we show that these habitat effects are the result of the behaviour and movement bias of frugivorous birds. By searching for fruit resources and the protection of forest cover, frugivores impose habitat-related spatial templates to the seed rain. In fact, the large-scale seed rain of all studied trees was strongly affected by the distribution of forest cover, irrespective of fruit abundance (Table 3). Moreover, the distribution of fruits of a given fleshy-fruited species may affect the seed rain of the others. For example, the seed rain of *C. monogyna* was related to the distribution of fruit crops, which were largely dominated by *I. aquifolium* (see also Figs. 2 and 3 in [21]).

### Advantages of a Behavioural-Based, Multi-Specific Model

Our modelling approach highlights the need to incorporate the landscape features that bias frugivore movement and hence seed dispersal. Focusing only on bird movement distances results in overly optimistic predictions, both in terms of dispersal distance and in seed arrival to open areas. For all the six species of *Turdus* followed in the field, we could detect movement biases due to forest cover and/or fruit abundance. However, we ran a “distance-only” model where we ignored such biases in order to provide a baseline showing the expected seed dispersal patterns resulting from the combination of gut passage time, perching time and displacement distances. In essence this is what several recent studies have done, combining animal movement data with seed retention time in order to estimate seed dispersal kernels (e.g. [22], [37], [38], [39], [40]. From the plants perspective, this model was more optimistic than that including biases due to forest cover and fruit abundance. Seeds were dispersed further, they were redistributed over space in a much more homogeneous fashion and many of them reached open landscape cells (Figs. 2 and 3). In contrast, the full model highlighted, in terms of dispersal kernels and seed rain patterns, the differences among the *Turdus* species, including the scale of their movement decisions and the relative importance of forest cover and fruit abundance in biasing their movements. Observed animal movement is the outcome of an interaction between the individual’s motivations and limitations and the structure of the landscape and any extrapolation will not be realistic unless these interactions are taken into account [3].

Interestingly, because different movement behaviours implied differences in space use, simulated bird species had slightly different diets even though they were modelled as generalists (Fig. 5). Also, as seed dispersal results from an interaction between bird behaviour (movement, perching and gut passage time) and landscape structure (the distribution of plants, cover and fruits), there were differences in mean dispersal distances for each combination of plant and bird species (Table 2). Those differences were more noticeable for the smaller bird species, *T. iliacus* and *T. philomelos*, which were the most sensitive to changes in forest cover (Fig. 1.E). In the case of rarer species (namely, *T. pilaris* and *T. torquatus*), our results could suggest a poorer fitting of model functions given we had less opportunities to collected data and to estimate parameters accurately. Nevertheless, there is empirical evidence suggesting that our fitted values are realistic. In our study site, we observed that *T. pilaris* appeared in small flocks that responded to forest cover but not to fruit abundance (Daniel Martínez unpublished data; 2010–11).Similarly, our analysis did not support the inclusion of fruit abundance in the movement model for this species. In the case of the rarest *T. torquatus* we observed solitary individuals usually at vantage points in open habitats with high visibility when feeding on fruit trees (authors’ pers. obs.).

Some of the differences among bird species were related to their body size. Besides the increase in gut retention time that we obtained from the literature, our observations showed that perching time also increased with body size (Fig. 1.B). Furthermore, the simulation results showed an increase in the probability of dispersing seeds out of the study plot and in the probability of seeds arriving at landscape cells without forest cover with body size (Figs. 2.B and F). Also, larger species included proportionally more *C. monogyna* and less *I. aquifolium* fruits in their diets, reflecting differences in space use (Fig. 5), leading us to suggest that behavioural responses of frugivores could translate into differences in plant-frugivore interactions [20], [41], [42].

### Caveats and Limitations

Several aspects of the seed dispersal process in our study site were left out of our current model and further work is needed in order to get a more accurate representation of the seed dispersal process and to assess the potential relevance of these “missing links”. Among the migrant species for example, some might arrive at different times in the season such that they could encounter different maps of fruit abundance depending on both plant phenology and previous fruit consumption by other resident or migrant species [43]. Thus, the relative abundance of thrushes is a dynamic property of the systems which might be relevant for seed dispersal. Also, despite the considerable area covered by our surveys, we found that many times the tracked birds flew out of the study plot. It seems that birds alternated between foraging moves within a site and longer displacements among forest patches. Previous work has pointed out that these thrushes are capable of large-scale resource tracking and often make long moves in flocks [30]. More detailed tracking techniques are needed for a better characterization of movement strategies and their potential impact on seed dispersal. Finally, in this exercise we have ignored fine-scale differences in seed depositions within a landscape cell [23],but this could be easily incorporated in further model developments.

### Concluding Remarks

By focusing on a straightforward, but ecologically relevant frugivore-plant assemblage, this study represents a first attempt to develop a mechanistic, community-based framework of seed dispersal by animals. We argue that our conclusions on how shared frugivores contribute to complementary seed dispersal and how fruiting landscapes generate community-level spatial fingerprints may be generalized to other temperate and tropical forest systems, especially those hosting functionally different frugivores able to track resources at different spatial scales (e.g. [16], [44], [45]).

Given that we could estimate the mechanisms underpinning the functional diversity within the frugivore guild, our modelling approach may be implemented to explore the link between the diversity of frugivores and the provision of seed dispersal service [33]. More importantly, our approach can be used to study potential changes in seed dispersal functionalities derived from shifts in species abundances or extinctions associated to global change. Finally, we show that the individual behavioural decisions of frugivores have the potential to affect the dynamics of a plant population or community. Further work is needed in order to follow the fate of dispersed seeds and eventually complete the link between bird behaviour and plant dynamics [5], [14].

## Supporting Information

Text S1 **Detailed description of the study system and field methodologies, including scheme of the field study plot showing forest cover and details of the sampling design.**
(DOCX)

Text S2 **Gut Passage Time estimation for Turdus sp. and seeds of fleshy-fruits in the Cantabrian Range.**
(DOCX)

Text S3 **Analysis for perching time and fruit consumption for the six** ***Turdus*** **species followed in the field.**
(DOCX)

Text S4 **Model fit, comparison and assessment for movement rules of** ***Turdus*** **species in the study plot.**
(DOCX)

Text S5 **Assessment of landscape-scale spatial structure of observed and predicted seed rain.**
(DOCX)
