# Supplementary material for: Frugivore Behavioural Details Matter for Seed Dispersal: A Multi-Species Model for Cantabrian Thrushes and Trees
Source: PLoS One. 2013 Jun 11;8(6):e65216. doi: 10.1371/journal.pone.0065216 (PMC3679117; doi:10.1371/journal.pone.0065216)

**Online Text S1**

# Detailed description of the study system and field methodologies

### Study system

We focused on the plant-frugivore assemblage composed of fleshy-fruited trees and frugivorous thrushes (*Turdus* spp.) in the temperate secondary-growth forest of the Cantabrian range (N Spain), a highly heterogeneous habitat resulting from strong anthropogenic impact. Temperate secondary forest is a common, but highly fragmented habitat type in mid-elevation areas of the Cantabrian range [1]. Typically, secondary forest stands cover less than 30% of surface in these areas, and may occur as fringe patches, adjacent to beech (*Fagus sylvatica*) stands. More frequently, they occur as variable-sized fragments (from isolated remnant trees to patches of several hectares) embedded in a historically deforested matrix of stony pastures and heathland (*Erica* spp., *Ulex europaeus*; [2]. These woodland-pasture areas are traditionally used for extensive cattle raising. Secondary forests are dominated by fleshy-fruited trees, mostly Holly (*Ilex aquifolium*), Hawthorn (*Crataegus monogyna*), Yew (*Taxus baccata*), which account for a large proportion of tree cover (ca. 70%, e.g. [3]. Fruits of these species are 10-14 mm diameter, sugar-rich red berries (arillated seed in yew), and contain 1-4 seeds (5-9 mm). All species ripen in autumn (September to November).

The fruits of the above tree species are consumed from trees by birds and, once fallen to the ground, by carnivorous mammals [4]. Among birds, the main frugivore species are thrushes (*Turdus* sp.) of increasing size (see Table S1.1 in Supporting information Appendix S1): Redwing *T.iliacus*, Song Thrush *T. philomelos*, Blackbird *Turdus merula,* Fieldfare *T. pilaris*, Ring-Ouzel *T. torquatus* and Mistle Thrush *T. viscivorus*. Among these species *T. iliacus,* *T. pilaris* and *T. torquatus* are over-wintering species in north-western Spain, whereas *T. philomelos*, *T. merula*, and *T. viscivorus* are resident species that receive overwintering migrants [5]. All thrushes are insectivorous birds whose diet turns to almost exclusively frugivore during autumn and winter. They swallow the entire fruits, defecating (and occasionally regurgitating) the intact seeds in their faeces and, thus, acting as legitimate seed dispersers.

Seed dispersal by thrushes has been shown to affect regeneration of fleshy-fruited trees in the Cantabrian secondary forest, by triggering the processes of re-colonization of deforested areas [6], and by driving the patterns of long-term recruitment at different spatial scales [3,7].

*Study site and spatio-temporal framework*

The study area was located in the Sierra de Peña Mayor (43º 17'N, 5º 30'W, 900 m a.s.l., Asturias Province, Spain), a mountain range covered by woodland pastures where secondary forest are intermingled with meadows, heathland and limestone rocky outcrops. There, in September 2006, we set up a 400 x 440 m rectangular plot in which the amount of forest cover varied from densely covered sectors to areas of scant cover and isolated remnant trees (see Fig. S1). Thus, the plot was chosen to represent a gradient of forest loss and increasing forest subdivision and forest edge [7]. For sampling purposes, the plot was subdivided into 440, 20 x 20 m cells. Based on previous findings [8], we assumed this spatial framework to have appropriate grain and extent sizes to represent the spatial scale at which frugivory and seed dispersal by thrushes operate. Sampling (see below) was carried out from September to February along three consecutive sampling periods: 2007-2008, 2008-2009 and 2009-2010 (hereafter 2007, 2008 and 2009).

*Forest cover and fruit abundance measures*

In September 2007, we developed a Geographical Information System (GIS, ArcGIS 9.0) based on a recent (2007) 1:5000-scale ortophotomap image of the study plot. The GIS platform incorporated a grid of 440, 20 x 20 m landscape cells, as well as a layer of digitized forest cover (Fig. S1.1). From this GIS we estimated the amount of forest cover (in m^2^, and irrespective of tree species identity) in each cell. We assumed that the per-cell values of forest cover measured in 2007 were also applicable to 2008 and 2009, as actual changes in tree canopy cover in the plot were negligible over years (canopy growth was undetectable at this resolution and tree-fall gaps were very scarce and small).

In autumn of each sampling year, we walked carefully all the study plot, identifying and mapping all individual trees (>1.5 m tall or 4 cm trunk basal diameter) of all species. We assigned visually a standing crop to each tree of any fleshy-fruited species, by means of a semi-quantitative Fruiting Abundance Index (FAI; considering six intervals: 0 = without fruits; 1 = 1-10 fruits; 2 = 11-100; 3 = 101-1,000; 4 = 1,001-10,000; 5 > 10,001). Fruiting is quite synchronous among individuals and species within the Cantabrian secondary forest, with almost all fruit ripening delimited to 1-2 months (although fruits may remain attached to trees for 1-3 additional months). We thus considered that a sampling of fruit abundance at the beginning of the season provided an appropriate estimate of the spatial template of fruit resources for frugivores through the season. As *T. baccata* show earlier fruiting peaks than the remaining fruiting species, we first walked the plot in late September to survey the crops of these species, and walked again the plot in late October, recording the fruit crops of all remaining fleshy-fruited species. All data on position, species and FAI of each individual tree, every sampling year, were incorporated into the GIS platform. We calculated the total abundance of fruits per cell, and the abundance of fruits of different tree species, per year as the sum of the crops of all fruiting trees present each year. Crops were extrapolated from FAI ranks, taking into account the fit between the actual crop size of a sub-sample of trees and the assigned FAI index, using an allometric equation (*crop size* = 1.77 × exp(1.92 FAI), *R^2^* = 0.80; *N* = 136 trees [9].

*Bird feeding activity and movement*

Foraging patterns of thrushes were sampled over observation sequences made from five different vantage positions in elevated outcrops (hill tops), located along the central axis of the plot (Fig. S1). Sampling season extended from October to February. Observation time was 78, 90 and 79 h for 2007, 2008 and 2009, respectively. In each sequence, a movement bout of a given individual bird was tracked with the help of 8 x 30 binoculars, a chronometer, and printed maps of plot cells. Once a given focal bird was located, it was followed until lost either because it left the plot or disappeared into the canopy). For all sequential steps in the movement bout (i.e. consecutive rests separated by intervening flights), we recorded the duration and location of the resting site (i.e. the cell within the plot), and the species and number of fleshy fruits eaten while perching in a fruiting tree. Flight distance was calculated for each flight between rests located in different cells as the Euclidean distance between the centroids of the starting point and endpoint cells.

*Sampling of seed dispersal by thrushes in the field*

Bird-dispersed tree seeds are unequivocally identifiable: they are clean of pulp remains, unlike seeds on fruits fallen beneath trees, and occur in small clusters easily distinguishable from those occurring in mammal faeces, and they can be almost exclusively attributable to thrushes [4]. We assessed the occurrence of seeds dispersed by thrushes in sampling stations across the whole plot in 2009. Ten sampling stations, separated from each other by 2 m, were placed along the central longitudinal axis of 220 sampling cells following a checkered pattern (see Fig. S1). In each station, we set up a permanently labelled, open-ground 50 x 50 cm quadrat where all tree seeds dispersed by birds were collected and counted. Each sampling station was assigned to covered (tree canopy) or open (pasture, rock) microhabitats, corresponding to the structural features of the area covered by (or above) the quadrat. We estimated the number of dispersed seeds per tree species sampling station as the sum of seeds found in two consecutive surveys (late November 2009 and early January 2010). Previous studies in the same site demonstrated that this methodology provides estimates of seed abundance and richness reliable enough for the evaluation of the large-scale patterns of seed dispersal [3,8]. In fact, seed removal by diurnal animals was never observed, and removal by nocturnal rodents is low during most of the dispersal season (predation frequency peaks late in winter, [10]. In any case, those seeds showing signs of predation (open husks or teeth marks) found in the quadrats were considered as dispersed seeds.

**References**

1. García D, Quevedo M, Obeso JR, Abajo A (2005) Fragmentation patterns and protection of montane forest in the Cantabrian range (NW Spain). Forest Ecology and Management 208.

2. Herrera JM, García D, Morales JM (2011) Matrix effects on plant-frugivore and plant-predator interactions in forest fragments. Landscape Ecology 26: 125-135.

3. García D, Obeso JR, Martinez I (2005) Spatial concordance between seed rain and seedling establishment in bird-dispersed trees: does scale matter? Journal of Ecology 93: 693-704.

4. Martínez I, García D, Ramon Obeso J (2008) Differential seed dispersal patterns generated by a common assemblage of vertebrate frugivores in three fleshy-fruited trees. Ecoscience 15.

5. Guitián J, Guitián P, Munilla I, Guitián J, Bermejo T, et al. (2000) Zorzales, espinos y serbales. Un estudio sobre el consumo de frutos silvestres de las aves migratorias en la costa occidental europea. Santiago de Compostela: Universidad de Santiago de Compostela.

6. García D, Martínez D, Herrera JM, Morales JM (2012) Functional heterogeneity in a plant–frugivore assemblage enhances seed dispersal resilience to habitat loss. Ecography in press.

7. Herrera JM, García D (2010) Effects of Forest Fragmentation on Seed Dispersal and Seedling Establishment in Ornithochorous Trees. Conservation Biology 24.

8. García D, Zamora R, Amico G (2011) The spatial scale of plant-animal interactions: effects of resource availability and habitat structure. Ecological Monographs 81: 123-139.

9. Herrera JM, Morales JM, García D (2011) Differential effects of fruit availability and habitat cover for frugivore-mediated seed dispersal in a heterogeneous landscape. Journal of Ecology 99: 1100-1107.

10. García D, Obeso JR, Martínez I (2005) Rodent seed predation promotes bird-dispersed trees in temperate differential recruitment among secondary forests. Oecologia 144.

**Figure S1.1.** Scheme of the field study plot representing: A) the configuration of the forest cover (grey area), the plot subdivision into 20x20 m landscape cells, and the vantage (black stars) positions for bird observation; B) a detail of the position of the stations for sampling seed deposition by frugivores (white squares) along the central longitudinal axis of cells following a checkered-pattern.


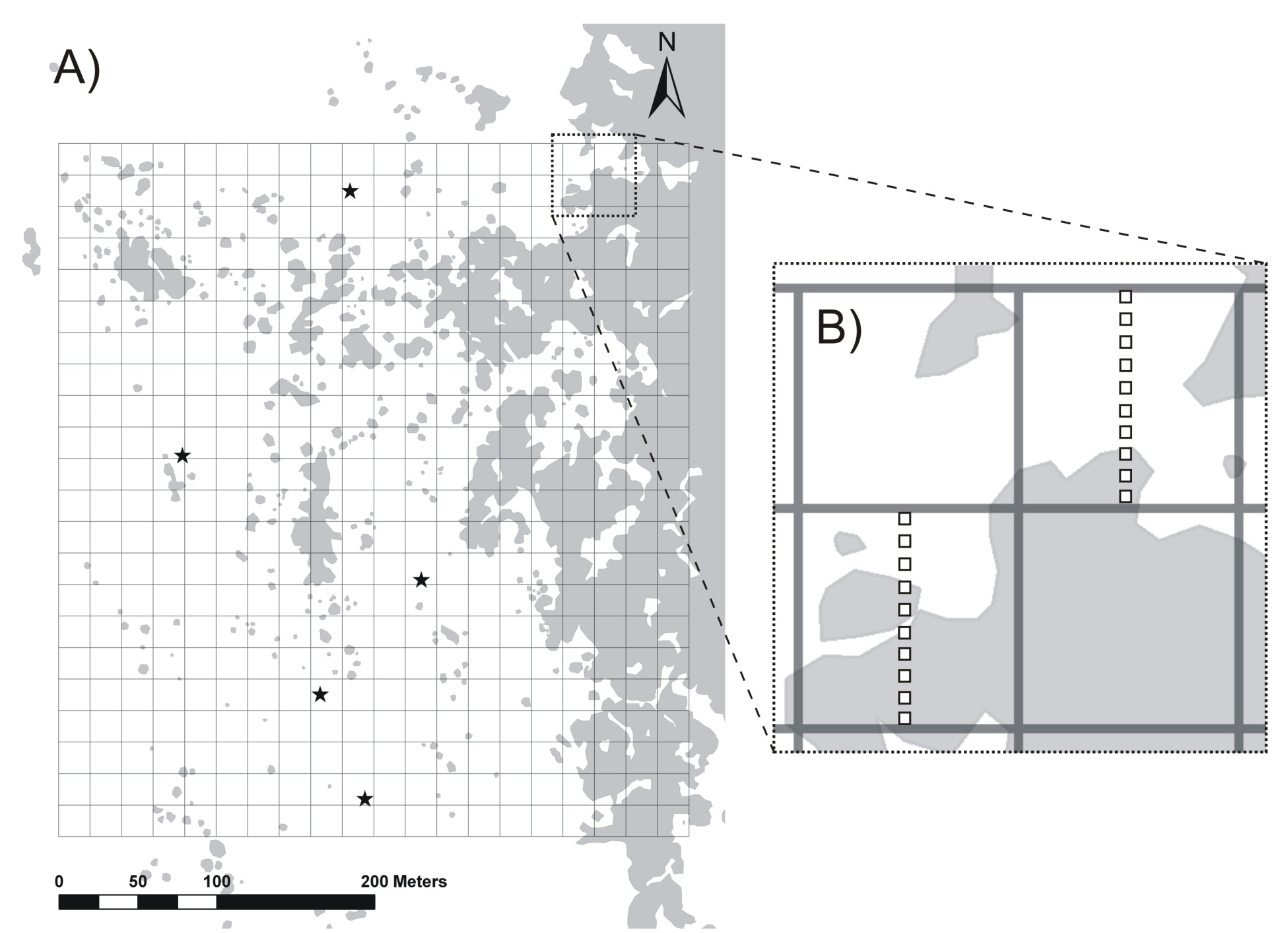

Supplement: Text S1 — Detailed description of the study system and field methodologies, including scheme of the field study plot showing forest cover and details of the sampling design. (DOCX) [file pone.0065216.s001.docx]
