# Supplementary material for: Frugivore Behavioural Details Matter for Seed Dispersal: A Multi-Species Model for Cantabrian Thrushes and Trees
Source: PLoS One. 2013 Jun 11;8(6):e65216. doi: 10.1371/journal.pone.0065216 (PMC3679117; doi:10.1371/journal.pone.0065216)
Supplement: Text S2 — Gut Passage Time estimation for Turdus sp. and seeds of fleshy-fruits in the Cantabrian Range. (DOCX) [file pone.0065216.s002.docx]

**Online Text S2**

**Gut Passage Time estimation for *Turdus* sp. and seeds of fleshy-fruits in the Cantabrian Rang**e

**Regurgitation *vs.* defecation**

We have calculated Gut Passage Time (GPT) distribution based exclusively on seed defecation times. Although seed regurgitation from fleshy-fruited species, like *Crataegus monogyna* and *Prunus avium*, has been observed (e.g. *Turdus merula* in captivity, Sorensen 1984; Breitbach et al. in press), we assumed that seed regurgitation of fleshy-fruited species by *Turdus* sp. is very rare in the Cantabrian Range, by the following reasons:

1. Sorensen’s finding was probably related to the much larger seed mass of *C. monogyna* fruits used in her study (130 mg in Soresen 1984 vs. 87 mg in García et al. 2005). Similarly, regurgitation of *P. avium* seeds was associated to its big size (fruit diameter > 15 mm), much bigger than those of the fleshy-fruited species studied here.
2. We had never observed regurgitation in the field (from aprox. 450 hours of direct observation on foraging sequences of individual birds of different species across 3 years). Similar results have been found in other studies in the same fruit-frugivore system in northern Spain (Guitián et al. 2002).
3. Regurgitation has been found to be rare (<5% of ingested seeds) in other captivity studies involving thrushes and fleshy fruits in the range of size of the studied here (*Turdus helleri* eating *Xymalos monospora*, Lehouck et al. 2009; *Turdus merula* eating *Crataegus monogyna*, Sobral et al. 2010; *Turdus merula* eating *Myrtus communis*, M. Sobral, A.R. Larrinaga & L. Santamaría unpublished)

**GPT values and distribution**

As no empirical data on the distribution of values of GPTs was available from any of the studied bird-fruit species pairs, we used data from a similar bird-fruit species pair: *Turdus merula* eating fruits of *Myrtus communis* (Mar Sobral, Asier R. Larrinaga & Luis Santamaría, unpublished data). GPT measures were done in January 2006 at the Institut für Vogelforshung, Vogelwarte Helgoland (Germany). Feeding trials in captivity conditions involved 18 hand-raised and wild *Turdus merula* individuals (see Sobral et al. 2010 for a comprehensive description of aviary conditions and bird care methodology in a similar study). GPT was measured as the time from swallowing to defecation per individual seeds. Average GPT resulted in 39.35 min. (±2.29SE; CV=173.98; N=890 seeds; Fig. S.1.1). This value was only slightly longer than that found by Breitbach et al. (in press) for seed regurgitation of *P. avium* seeds by *T. merula* (median 29.6 min).

Given the similarity of fruits of *Crataegus monogyna*, *Ilex aquifolium* and *Taxus baccata* in the Cantabrian range (in terms of fruit mass; seed load; pulp to seed ratio) we assumed that average values and distribution of frequencies of GPTs were equivalent between tree species for *T. merula*, and similar to that found in *Myrtus communis*. In fact, no relationship between fruit or seed size and GPT across species has been found for *Turdus merula* (Sorensen 1984). We assumed that in *Ilex aquifolium*, all seeds per fruit (average 3.5 seeds/fruit) were defecated at the same time (the average number of seeds per dropping defecated in the field is 3.4; Martínez et al. 2008; see also Guitián et al. 2002).

**Figure S2.1.** Frequency distribution of gut passage time for *Myrtus communis* seeds defecated by *Turdus merula*.


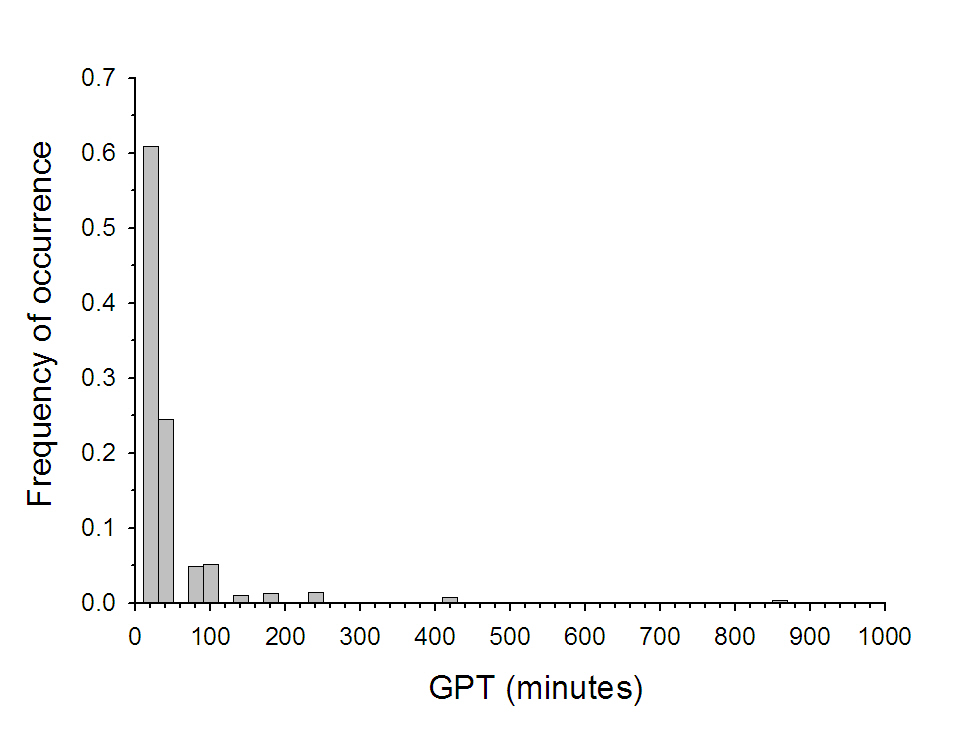


**Extrapolation from *Turdus merula* to other *Turdus* spp. Relationship between bird body size and GPT:**

We calculated a body size-GPT relationship from Herrera (1984) using 8 species from Turdidae and Silvidae, and the GPT of a Barium Sulfate solution:

GPT = 23.73 + 0.51*weight

Slope: 0.511±0.14

Linear regression R^2^ = 0.68; F_1,6_ = 12.74; P = 0.012

Using the slope of this relationship, we calculated an intercept of -11.76 corresponding to a GPT of 39.35 min (which is the average GPT value for *Myrtus communis* seeds for *Turdus merula* in captivity, Fig. 1). Thus, the relationship between the *Turdus* body size and GPT for any fleshy-fruit species should be based on the relationship

GPT = -11.76 + 0.511*weight (0.1)

Thus, to extrapolate GPT for species other than *Turdus merula*, we used GPT distribution of *Myrtus communis* in *Turdus merula* and transform GPT data by using the proportions between species obtained from GPT in eq. (0.1) given the coefficients from Table 1. For example, a GPT=15 min in *Turdus merula* would correspond to 8.17 (=15*0.545) min in *Turdus iliacus*, 16.93 min in *Turdus pilaris*, 10.15 min in *Turdus philomelos*, etc.

**Table S2.1.** Coefficients of GPT for different species of thrushes based on body size and refereed to *Turdus merula* GPT.

| Species | Body size (g)* | mean GPT estimate | Rate for Gamma distribution† |
| --- | --- | --- | --- |
| *T. iliacus* | 65 | 21.45 | 0.074 |
| *T. pilaris* | 110 | 44.45 | 0.0357 |
| *T. merula* | 100 | 39.34 | 0.04 |
| *T. philomelos* | 75 | 26.57 | 0.0598 |
| *T. torquatus* | 120 | 49.56 | 0.032 |
| *T. viscivorus* | 130 | 54.67 | 0.029 |

* from Collar (2005)

† assuming the shape parameter is 1.59 as in the Gamma distribution fitted to the data from *Turdus merula* above.

**References**

Breitbach, N, Böhning-Gaese, K., Laube., I. and Schleuning, M. In press. Short seed-dispersal distances and low seedling recruitment in farmland populations of bird-dispersed cherry trees. Journal of Ecology. doi: 10.1111/1365-2745.12001

Collar, N. J. (2005) Family Turdidae (Thrushes). Pp. 514-807 in del Hoyo, J., Elliot, A. & Christie, D.A. eds. (2005) Handbook of the Birds of the World. Vol. 10. Cuckoo-shrikes to Thrushes. Lynx Edicions, Barcelona.

García D., Obeso, J. R. & Martínez, I. 2005. Rodent seed predation promotes differential seedling recruitment among bird-dispersed trees in temperate secondary forests. Oecologia 144: 435-446

Guitián, J., Guitián, P., Munilla, I., Guitián, J., Bermejo, T., Larrinaga, A. R., Navarro, L., and López, B. 2000. Zorzales, espinos y serbales. Un estudio sobre el consumo de frutos silvestres de las aves migratorias en la costa occidental europea. Universidad de Santiago de Compostela, Santiago de Compostela.

Herrera, C. M.1984. Adaptation to frugivory of Mediterranean avian seed dispersers. Ecology 65: 609-617.

Lehouck, V., Spanhove, T., Demeter, S., Groot, N. E., and Lens. L. 2009. Complementary seed dispersal by three avian frugivores in a fragmented Afromontane forest. Journal of Vegetation Science 20: 1110–1120.

Martínez, I., García, D. & Obeso, J. R. 2008. Differential seed dispersal patterns generated by a common assemblage of vertebrate frugivores in three fleshyfruited trees. Ecoscience 15: 189–199.

Sobral, M., Larrinaga, A. R., and Guitián, J. 2010. Fruit preferences in wild and naïve blackbirds (*Turdus merula*) feeding on hawthorn fruits (*Crataegus monogyna*). The Auk 127: 532-539.

Sorensen, A. 1984. Nutrition, Energy and Passage Time: Experiments with Fruit Preference in European Blackbirds (*Turdus merula*). Journal of Animal Ecology 53: 545-557.
