# Supplementary material for: Frugivore Behavioural Details Matter for Seed Dispersal: A Multi-Species Model for Cantabrian Thrushes and Trees
Source: PLoS One. 2013 Jun 11;8(6):e65216. doi: 10.1371/journal.pone.0065216 (PMC3679117; doi:10.1371/journal.pone.0065216)
Supplement: Text S3 — Analysis for perching time and fruit consumption for the six Turdus species followed in the field. (DOCX) [file pone.0065216.s003.docx]

Online Text S3

**Analysis for perching time and fruit consumption for the six *Turdus* species followed in the field.**

Figure S3.1. Observed and fitted distributions of time perching for the six *Turdus* species.


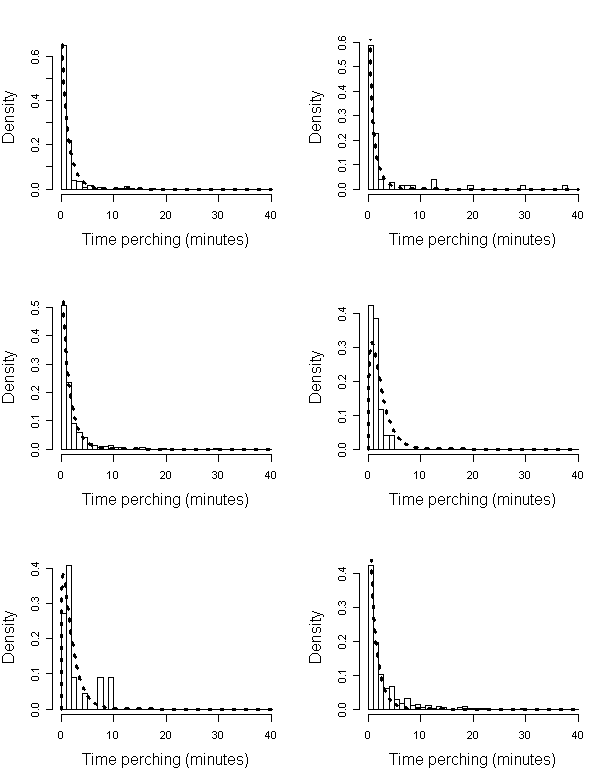


*Turdus viscivorus*

*Turdus torquatus*

*Turdus pilaris*

*Turdus philomelos*

*Turdus merula*

*Turdus iliacus*

Table S3.1. Parameters with SE in parenthesis for Gamma distributions of time perching at plants or standing on the ground

|  | shape | rate | Expected (min) | Median (min) | N |
| --- | --- | --- | --- | --- | --- |
| *Turdus iliacus* | 0.849 (0.067) | 0.638 (0.067) | 1.331 | 0.633 | 244 |
| *Turdus philomelos* | 0.569 (0.077) | 0.204 (0.042) | 2.796 | 0.833 | 75 |
| *Turdus merula* | 0.931 (0.054) | 0.492 (0.037) | 1.891 | 1.000 | 458 |
| *Turdus pilaris* | 1.472 (0.371) | 1.114 (0.334) | 1.322 | 1.166 | 26 |
| *Turdus torquatus* | 1.256 (0.339) | 0.435 (0.144) | 2.883 | 1.708 | 22 |
| *Turdus viscivorus* | 0.747 (0.044) | 0.269 (0.022) | 2.773 | 1.416 | 427 |

Table S3.2. Pearson's product-moment correlations between perching time and fruit consumption for all species

|  | r |  | t and df | p - value | CI |
| --- | --- | --- | --- | --- | --- |
| *Turdus iliacus* | 0.131 |  | t = 1.743, df = 175 | 0.0831 | -0.017 0.273 |
| *Turdus merula* | -0.009 |  | t = -0.1648, df = 284 | 0.8692 | -0.126 0.106 |
| *Turdus philomelos* | 0.200 |  | t = 1.4582, df = 51 | 0.1509 | -0.074 0.446 |
| *Turdus pilatus* | 0.369 |  | t = 1.5894, df = 16 | 0.1315 | -0.118 0.713 |
| *Turdus torquatus* | -0.224 |  | t = -0.6899, df = 9 | 0.5077 | -0.726 0.434 |
| *Turdus viscivorus* | 0.076 |  | t = 0.9586, df = 158 | 0.3392 | -0.080 0.229 |

Table S3.3. Zero –inflated Poisson model for fruit consumption. For each species we estimated the probability of consuming fruits and the mean of a Poisson distribution (Lambda). Confidence intervals based on profile Likelihoods are reported in parenthesis.

|  | Probability of  fruit consumption | Average number of fruits  per feeding bout | N |
| --- | --- | --- | --- |
| *Turdus iliacus* | 0.531 (0.458 – 0.603) | 5.168 (4.725 – 5.639) | 179 |
| *Turdus philomelos* | 0.370 (0.249 – 0.503) | 6.560 (5.530 – 7.791) | 54 |
| *Turdus merula* | 0.524 (0.467 – 0.580) | 5.916 (5.541 – 6.307) | 296 |
| *Turdus pilaris* | 0.500 (0.281 – 0.719) | 6.556 (5.022 – 8.373) | 18 |
| *Turdus torquatus* | 0.455 (0.194 – 0.735) | 5.560 (3.773 – 7.937) | 11 |
| *Turdus viscivorus* | 0.335 (0.266 – 0.409) | 5.145 (4.569 – 5.768) | 164 |
