# Supplementary material for: Frugivore Behavioural Details Matter for Seed Dispersal: A Multi-Species Model for Cantabrian Thrushes and Trees
Source: PLoS One. 2013 Jun 11;8(6):e65216. doi: 10.1371/journal.pone.0065216 (PMC3679117; doi:10.1371/journal.pone.0065216)

Online Text S4

**Model fit, comparison and assessment for movement rules of *Turdus* species in the study plot**

Table S4.1

Parameter values and standard errors for the movement models (equations 1 and 2 in the main text) of the six *Turdus* species followed in the field.

|  | *T. iliacus* | *T. philomelos* | *T. merula* | *T.pilaris* | *T. torquatus* | *T. viscivorus* |
| --- | --- | --- | --- | --- | --- | --- |
| *a_d_* | -4.407 (0.135) | -3.717 (0.228) | -4.093 (0.079) | -3.669 (0.400) | -3.662 (0.434) | -3.905 (0.107) |
| *b_d_* | -0.647 (0.0608) | -0.398 (0.114) | -0.437 (0.046) | -0.406 (0.212) | -0.242 ( 0.293) | -0.517 (0.050) |
| *a_c_* | 1.082 (0.798) | 0.746 (0.372) | 0.252 (0.025) | 0.269 (0.134) |  | 0.192 (0.049) |
| *b_c_* | 1.397 (0.304) | 1.762 (0.469) | 3.278 (0.634) | 2.382 (1.457) |  | 1.477 (0.547) |
| *a_f_* | 16.833 (100.907) | 3.577 (16.023) | 2.582 (6.762) |  | 0.105 ( 0.003) | 0.0978 (0.018) |
| *b_f_* | 0.601 (0.155) | 0.847 (0.265) | 0.335 (0.114) |  | 50.119 (28.732) | 2.198 (4.194) |
| *a_o_* | -0.073 (0.523) | -1.429 (0.842) | -1.078 (0.382) | 0.361 (1.503) | 0.714 (1.739) | -0.698 (0.2689) |
| *b_o_* | -3.718 (0.901) | -1.361 (0.991) | -1.752 (0.438) | -3.527 (2.732) | -2.643 (2.303) | -0.885 (0.291) |

Table S4.2 Delta AICc for all models and species

|  | *T. iliacus* | *T. merula* | *T. viscivorus* | *T. philomelos* | *T. pilaris* | *T. torquatus* |
| --- | --- | --- | --- | --- | --- | --- |
| distance, cover and fruit | 0 | 1.7 | 0 | 0 | 8.8 | 11.4 |
| distance and cover | 5.7 | 0 | 29.8 | 2.2 | 0 | 4.1 |
| distance and fruit | 56.1 | 103.4 | 29.8 | 22.2 | 4.6 | 0 |
| distance only | 121.4 | 166.4 | 25.7 | 62.2 | 3.5 | 2.5 |

Figure S4.1. quantile-quantile plots of distance moved for observed and simulated thrushes.


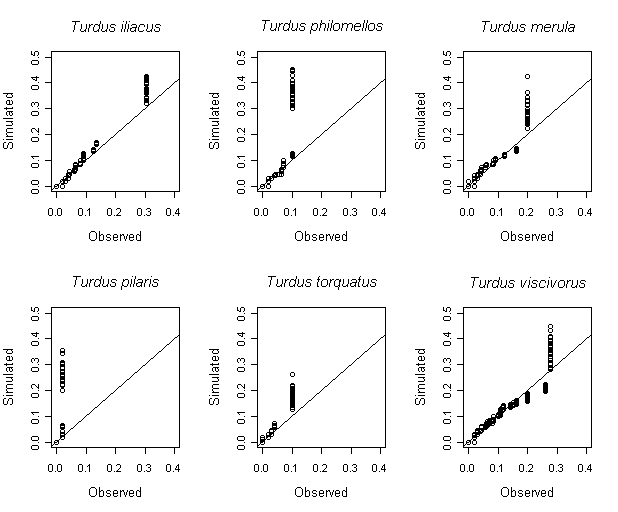

Supplement: Text S4 — Model fit, comparison and assessment for movement rules of Turdus species in the study plot. (DOCX) [file pone.0065216.s004.docx]
