# Supplementary material for: Frugivore Behavioural Details Matter for Seed Dispersal: A Multi-Species Model for Cantabrian Thrushes and Trees
Source: PLoS One. 2013 Jun 11;8(6):e65216. doi: 10.1371/journal.pone.0065216 (PMC3679117; doi:10.1371/journal.pone.0065216)
Supplement: Text S5 — Assessment of landscape-scale spatial structure of observed and predicted seed rain. (DOCX) [file pone.0065216.s005.docx]

**Online Text S5**

**Assessment of landscape-scale spatial structure of observed and predicted seed rain.**

*Spatial aggregation of the seed rain*

We characterized the landscape-scale spatial structure of predicted and observed seed rain of the different tree species (*Crataegus monogyna*, *Ilex aquifolium* and *Taxus baccata*) by quantifying the degree of spatial aggregation (patchiness) in the abundance of dispersed seeds. For that, we used the Spatial Analysis by Distance Indices (SADIE; Perry 1995; Perry et al. 1999), a method that describes the spatial structure of ecological data sampled in the form of spatially geo-referenced counts, identifying and locating the areas where patches of high- or low density occur.

We estimated, for the cell subset of field sampling (N=220), the abundance of dispersed seeds per cell observed in the field (from the average number of seeds per sampling station), and the corresponding abundance predicted by the model, for each tree species. Seed counts were referenced in space by the x,y coordinates of cell centroids. Based on estimates of distance to regularity (i.e. the difference between the true count value in a given point and a count value assuming a regular distribution of all counts across sampling points), SADIE provides an aggregation index (*Ia*) to measure the degree of overall spatial clumpiness across the whole plot extent, representing random (*Ia* = 1), regular (*Ia* < 1) or aggregated (*Ia* > 1) distribution patterns. The degree of significance of *Ia* is checked by means of a randomization procedure based on rearrangements of the observed counts amongst the sample units. SADIE also provides a point-level parameter, the clustering index (υ), which quantifies the degree to which the count at a given point contributes to the overall clumpiness. Points with high positive υ values contain big counts which contribute greatly to the generation of high-density clusters (“patches”), whereas points with negative υ values contain small counts which contribute to low-density clusters (“gaps”; e.g. Figure S5.1).

The aggregation indexes of both observed and predicted abundances of dispersed seeds at the landscape scale indicated strong and significant patchiness, with higher values on predicted seed rain than of observed seed rain, for all species (Table S5.1). Aggregation was stronger on *I. aquifolium* seed rain than on those of *C. monogyna* and *T. baccata*, in both the observed and predicted estimates.

**Table S5.1.** Indexes of aggregation (*Ia*) quantifying the degree of non-random patchiness in the seed rain of different tree species, both that observed in the field (average number of seeds per m2 per cell, 2009 data; n=220 cells) and that predicted by the model (number of seeds per cell, 2009 data; n=220 cells), with their associated significance degree (***: P < 0.001).

| Species | Observed seed rain | Predicted seed rain |
| --- | --- | --- |
| *Crataegus monogyna* | 1.83 *** | 2.42 *** |
| *Ilex aquifolium* | 3.62 *** | 3.93 *** |
| *Taxus baccata* | 1.96 *** | 3.68 *** |

**Figure S5.1.** Distribution of the abundance of dispersed seeds of different tree species across the study plot. Grey-scaled lined contours represent the SADIE-provided clustering indexes (υ) for of observed and predicted seed rain. Note the different scales between species.


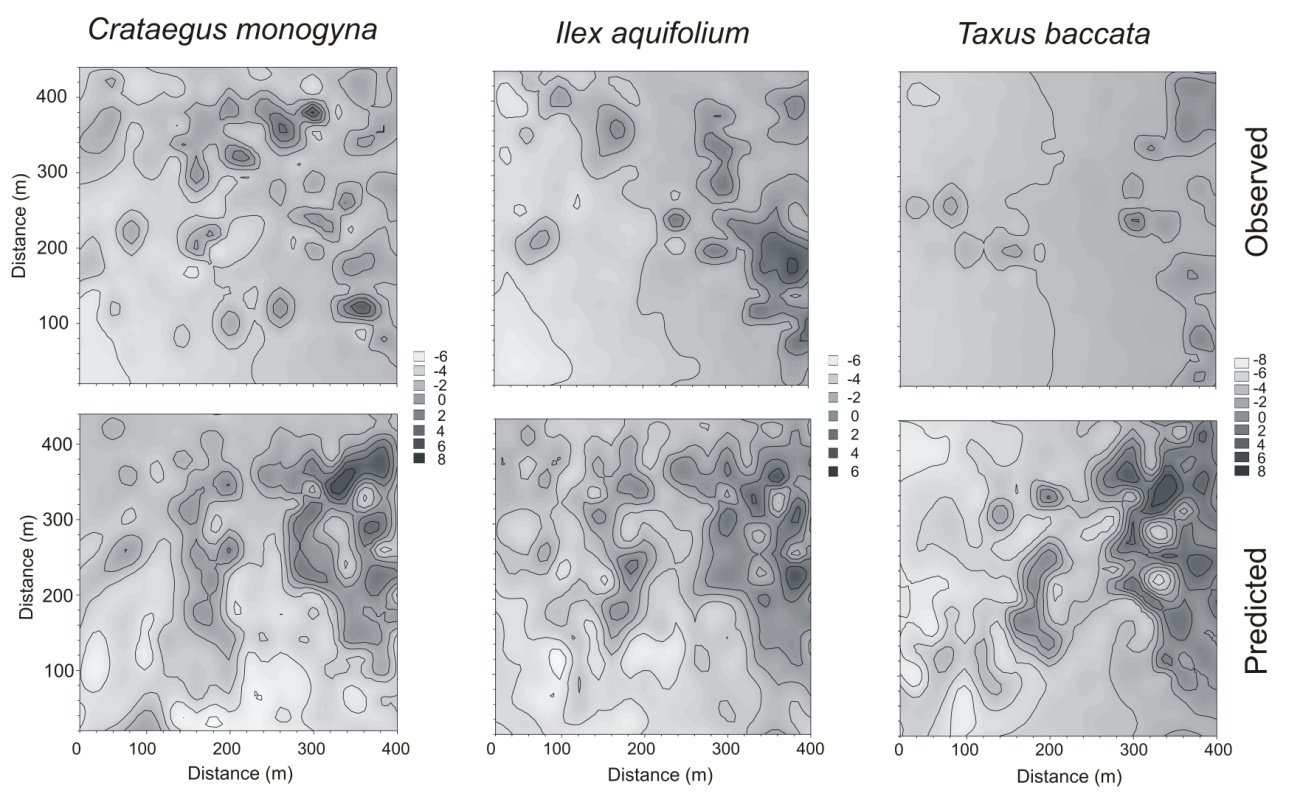


S*patial match between observed and predicted seed rain*

We also used SADIE to estimate the match between the spatial distributions of observed and predicted seed rains, in order to assess the ability of model-inferred seed rain to predict the large-scale spatial structure of seed dispersal in the field. For that, we related the clustering vectors of the observed seed rain with those of the seed rain predicted by the model in the same cells. Analyses relating clustering vectors must account for the potential effects of spatial autocorrelation in the determination of correlation strength. In this sense, the association index *X_p,_* (Perry & Dixon 2002) quantifies the degree of spatial association/dissociation between two variables sampled at the same points. This index ranges between + 1 (complete spatial association) and -1 (complete dissociation), with 0 indicating spatial independence. The statistical significance of *X_p_* is quantified by the Dutilleul method, which corrects the amount of degrees of freedom in the presence of spatial autocorrelation. Complementarily, in order to estimate the proportion of the variance in the patchiness of the observed seed rain explained by that predicted by the model, we regressed the clustering indexes of observed (response) and predicted (predictor) seed rain by means of Spatial Simultaneous Autoregressive Models (SAR, Keitt et al. 2002).

The seed rain predicted by our mechanistic model reproduced quite well the landscape scale spatial structure of observed seed rain. For all study tree species, there was a positive and significant spatial match between the landscape-scale patchiness of observed seed rain and that of seed rain predicted by the model, increasing from *C. monogyna* to *I. aquifolium* and *T. baccata* (Fig. 1; Table S5.2). In the last species, predicted seed rain accounted for 45% of the variance of observed seed rain.

**Table S5.2.** Spatial match between observed and predicted seed rains. The indexes of association (*Xp*), measuring the correlation between clustering indexes of observed and predicted abundance of dispersed seeds (n=220 cells), and their corresponding significance levels (corrected by Dutilleul’s method) are shown. Results of SAR considering the clustering indexes of observed seed rain as a response variable and those of predicted seed rain as predictor are also shown (R^2^ indicates the proportion of variance explained by predictor, without space effect).

| Species | *Xp* | Corrected N | P | R^2^* | t-value | P |
| --- | --- | --- | --- | --- | --- | --- |
| *Crataegus monogyna* | 0.356 | 198.8 | <0.001 | 0. 119 | 4.56 | <0.001 |
| *Ilex aquifolium* | 0.596 | 173.8 | <0.001 | 0.324 | 8.37 | <0.001 |
| *Taxus baccata* | 0.695 | 189.5 | <0.001 | 0.452 | 10.25 | <0.001 |

**References**

Keitt, T. H., Bøjornstad, O. N., Dixon, P. M. & Citron-Pousty, S. 2002 Accounting for spatial pattern when modelling organism-environment interactions. Ecography 25:616-625.

Perry, J. N. 1995. Spatial analysis by distance index. Journal of Animal Ecology 64:303-314.

Perry, J. N., Winder, L., Holland, J. M. and Alston R. D. 1999. Red-blue plots for detecting clusters in count data. Ecology Letters. 2:106-113.

Perry, J. N. & Dixon, P.M. 2002. A new method to measure spatial association for ecological count data. Ecoscience 9:133–141.
